# The Addition of Resveratrol-Loaded Emulsions to Yogurts: Physicochemical Characterization, In Vitro Bioaccessibility and NMR-Based Nutritional Profiles

**DOI:** 10.3390/foods13030426

**Published:** 2024-01-28

**Authors:** Zihui Shi, Huan Chen, Junbo He, Weinong Zhang, Hong Lin

**Affiliations:** 1 College of Food Science and Engineering, Wuhan Polytechnic University, Wuhan 430023, China; 15549080203@163.com (Z.S.); junb112he@whpu.edu.cn (J.H.);; 2 Key Laboratory for Deep Processing of Major Grain and Oil, Ministry of Education, Wuhan 430023, China

**Keywords:** resveratrol, sodium caseinate, composite nano-emulsions, bioaccessibility, NMR, yogurt

## Abstract

In this study, resveratrol-loaded nano-emulsions were added to yogurts, improving the physicochemical properties and functional factors and realizing the development of nutrient-fortified yogurt. Yogurts added with free resveratrol (Y-R), resveratrol-loaded emulsions stabilized by sodium caseinate (Y-NN), decaglycerol monooleate (Y-DN), and sodium caseinate-decaglycerol monooleate (Y-DND) were evaluated for their physicochemical properties, including pH, titratable acidity, syneresis, and textural parameters, with 5-day intervals for 15-day storage. The resveratrol retention rate was analyzed in the Y-R, Y-NN, Y-DN, and Y-NDN groups during 15 days of storage. The dynamic bioaccessibility of resveratrol and the NMR-based nutritional profile of yogurt in the Y-R, Y-NN, Y-DN, and the Y-NDN group were investigated after in vitro digestion. The results demonstrated that the addition of resveratrol emulsion decreased the hardness of yogurt while evaluating its titratable acidity and water-holding capacity, which were characterized by high stability. The stability of resveratrol added in the form of an emulsion was significantly higher than that of the free form. Compared with the other groups, the yogurt formulated with sodium caseinate/decaglycerol monooleate (NaCas/DGMO) emulsion showed the highest resveratrol retention rate, about 70%. In vitro digestion showed that encapsulation effectively and persistently improved the dynamic bioaccessibility of resveratrol. Additionally, NMR-based nutritional profile analysis before and after in vitro digestion demonstrated that resveratrol emulsion nutritional fortification promoted the release of nutrients, improving the nutritional value of yogurt. These findings offered theoretical guidance and technical support for the use of resveratrol nano-emulsions in yogurt.

## 1. Introduction

Yogurt is a traditional fermented dairy food. It has been widely favored by consumers over the years since it provides protein, peptides, prebiotics, and probiotics, which have multiple health benefits for the human body. Some studies have shown a correlation between yogurt intake and the prevention of heart disease. Regular intake of yogurt can reduce the risk of cardiovascular disease in adult hypertensive patients and type 2 diabetes. However, yogurts contain few active compounds. Obviously, nutritionally diversified yogurt will be more preferred by consumers and will have a larger market space. Therefore, adding natural active substances to yogurt and developing nutrition-enhanced yogurt is an important way to upgrade the yogurt industry. Resveratrol is a natural phytoalexin that originates from grapes, peanuts, and red wine. A number of in vitro and in vivo studies described positive biological impacts of resveratrol, such as antioxidative, anti-inflammatory, anticancer, and chemo-preventive activities [1,2]. However, structural instability to light and oxygen and poor solubility limited its application in food and beverages. Currently, various formulations are used to promote the oxidative stability and solubility of resveratrol, such as nano-structured lipid carriers (NLCs) [3], protein-carbohydrate Maillard conjugation [4], sodium caseinate [5], micelle/hydrogel composite [6], and emulsion [7]. Qayyum Shehzad et al. improved the oxidation stability of fish oil nano-lotion by con-encapsulating curcumin and resveratrol [8]. Decheng Bi et al. prepared an O/W nano-emulsion by loading resveratrol with unsaturated guluronate oligosaccharide (GOS) [9]. Among these, nano-encapsulation has been a promising strategy to address the abovementioned limitations. Studies involving these carriers of resveratrol have mainly focused on the evaluation of their stability [10], processability, in vitro digestive behaviors and release, while few resveratrol-loaded nano-particles have applied them to real food systems. 

The nano-encapsulation of bioactive ingredients has been applied to yogurts for years. Tahere Ghorbanzade established the nano-encapsulation of fish oil in nano-liposomes and used it in the fortification of yogurt [11]. Camila de Campoa [12] reported the application of zeaxanthin nano-particles in yogurt, improving the physicochemical properties and carotenoid release of yogurts. Recently, several plant components have been reportedly added to yogurts; for example, the juices from grapes and berries [13], saffron floral extracts [14], perilla seed oil [15], carrao (*Cassia grandis* L.) [16], dietary fiber from grapefruit [17], and taro starch (*Colocasia esculenta*) [18]. Researchers evaluated the impacts of these plant components on the physicochemical properties (including pH, acidity, color, sensory scores, and syneresis) and textural parameters (like hardness and viscosity), and found that they were essential for the market acceptance and shelf life of yogurt products. 

Although researchers have explored the potential of resveratrol in mitigating advanced glycation end-products formed in baked milk and baked yogurt [19], the resveratrol in that study was applied as a food additive to improve the processability. However, the application of resveratrol emulsions to fortified yogurt was limited; therefore, it is important to evaluate the effect of resveratrol-loaded nano-emulsions on the physicochemical properties and textural parameters of yogurt. Therefore, the resveratrol bioaccessibility and impact of the addition of resveratrol-loaded emulsions on the nutritional profile deserve further research. 

In vitro digestion is commonly considered an effective method to simulate in vivo digestion in humans. It is straightforward, low in cost, and has no ethical restrictions [20]. The dynamic bioaccessibility of active substances and their nutritional properties during in vitro digestion are easy to assess. Different analytical technologies can be used to describe the nutritional profile of food, for instance, high-performance liquid chromatography tandem mass spectrometry (HPLC-MS) and nuclear magnetic resonance (NMR). The NMR-based analysis of food components facilitates the comprehensive, non-destructive, and unbiased detection of small-molecule nutrients in foodstuffs. The NMR-based nutritional analysis of in vitro digestion has been reported in plant-based fishball analogue [21], abalone [22], and cold-stored strawberry [23]. 

In our previous work, we optimized the preparation of NaCas/DGMO composite nano-particles and established stable emulsions using these nano-particles [24]. It was found that the emulsions were stabilized under different complex processing conditions. Therefore, it was suitable for applications in food. In the present study, resveratrol-loaded emulsions were employed in yogurts. The influence of resveratrol with different formulations on the physicochemical properties, including pH, titratable acidity, water-holding capacity, and retention rate of resveratrol, was evaluated. Additionally, the bioaccessibility of in vitro resveratrol and NMR-based nutritional profiles were also investigated.

## 2. Materials and Methods

### 2.1. Materials and Reagents

Resveratrol and sodium caseinate (analytical grade) were purchased from Sigma-Aldrich China Co., Ltd. (Sigma-Aldrich, Shanghai, China). Polyglycerol fatty acid ester (food grade) was purchased from Shandong Binzhou Jinsheng New Material Technology Co., Ltd. (Binzhou, China). Rice bran oil (Jinlongyu, Shenzhen, China) was purchased from the local market. Acetonitrile and methanol (chromatographic grade) were purchased from Sinopharm Chemical Reagent Co., Ltd. (Shenzhen, China). The enzymes, including porcine gastric mucosal pepsin, pancreatic enzymes, and porcine bile extracts, were bought from Sigma-Aldrich (Sigma-Aldrich, Shanghai, China).

### 2.2. Preparation of Resveratrol-Loaded Emulsion

The preparation of a resveratrol-loaded emulsion was based on our previous work [24]. Briefly, the NaCas/DGMO complex (*w*/*w* = 1) dispersion was obtained by mixing NaCas and DGMO stock solutions for 1 h at 25 °C. For the preparation of an oily phase, 1 mL of 22 mg/mL of the resveratrol solution (in ethanol) was added to 4 g of rice bran oil. The mixture was subjected to stirring for 30 min at 85 °C to completely evaporate the ethanol. To obtain an emulsion with a final concentration of 0.5 mg/mL resveratrol, the oily phase was mixed with a 39.6 mL NaCas/DGMO complex (*w*/*w* = 1) dispersion and homogenized at 8000 rpm for 1 min, utilizing a high-speed blender (XHF-DY, Ningbo Xinzhi Tech. Co., Ltd. Ningbo, China). Following this, the resulting emulsion was subjected to three additional homogenization cycles with a Nano DeBEE30-4 high-pressure homogenizer (Atkinson, NH, USA) operating at a pressure of 10,000 psi. 

The corresponding control groups with equivalent resveratrol in water, NaCas-oil, and DGMO-oil dispersion were also prepared. The particle size and polydispersity index (PDI) of the complex dispersions were measured using a Malvern Zetasizer Nano ZS particle size analyzer (Malvern, UK). The samples were diluted 10 times. Then, the samples were characterized at 25 °C after being maintained for 300 s in the measuring chamber. Each sample was measured five times. Additionally, the emulsions were examined under a microscope (Olympus, Tokyo, Japan) using laser confocal microscopy (LSCM). A total of 1 mL of the emulsion was stained with 20 μL of the Nile red solution (1 mg/mL), vortexed for 5 min, and then protected from light for 12 h. A total of 10 μL of the stained emulsion was evenly spread on a slide, covered, and observed under a microscope at 100× magnification.

### 2.3. Preparation of Yogurts

The 100 g yogurt samples (Youzhiyou Dairy, Wuhan, China) were mixed separately with 15 mL of resveratrol in different formulations, including water (denoted by Y-R), NaCas-oil dispersion (Y-NN), DGMO-oil dispersion (Y-DN), and NaCas/DGMO-oil emulsion (Y-NDN). The compositions of five formulated yogurts are presented in Table 1. These mixtures were then stored in glass containers at 4 °C in a refrigerator for subsequent study. The physical–chemical characteristics and retention rates of the resveratrol were investigated every five days over 15 days at 4 °C.

### 2.4. In Vitro Gastrointestinal Digestion

In vitro gastrointestinal digestion was conducted based on the previously reported literature with slight modifications [25,26]. 

The enzyme activities were experimentally determined, as described by the INFOGEST protocol [27]. The activities of pepsin and trypsin in the final digest were 2000 U·mL^−1^ and 200 U·mL^−1^, respectively. The procedure was initiated with a gastric phase, followed by an intestinal phase. For the gastric phase, 10 μL of CaCl_2_ (0.3 mol/L) was mixed with 2.69 mL of distilled water and 16 mL of the porcine pepsin solution. The pH was adjusted to 3 using 1.3 mL HCl (1 mol/L). After preheating to 37 °C, 1 mL of SGF was mixed with 50 mg of each preheated sample. Then, they were incubated at 37 °C for 2 h in darkness while being stirred at 200 rpm. For the intestinal phase, 80 μL of CaCl_2_ (0.3 mol/L) was mixed with 6.96 mL of distilled water and 32 mL of pancreatin. Then, 0.3536 g of bile salts and 160 μL of amyloglucosidase were added and preheated to 37 °C. The pH of the pre-digestion solution was adjusted to 7 using a pH meter (F20, Mettler Toledo, Shanghai, China) with 800 μL NaOH (1 mol/L). The stomach sample was then combined with 2 mL of the prepared mixture, which was then digested in a water bath for two hours at 37 °C while being shaken at 200 rpm.

### 2.5. Sample Preparation for NMR Analysis

Yogurts after in vitro digestion were centrifuged at 5000 rpm for 5 min at 4 °C. The supernatant was lyophilized for further analysis. The blank in vitro gastrointestinal digestive fluid was processed using the same procedure as that used for the control. 

The freeze-dried sample (50 mg) was mixed with 1.5 mL of D_2_O and 0.01% sodium 3-trimethylsilyl [2,2,3,3-d4] propionate (TSP), centrifuged at 12,000 rpm for 10 min (4 °C), and then the obtained supernatant (600 μL) was transferred into a 5 mm NMR tube for analysis.

### 2.6. Determination of pH and Acidity of Yogurt Samples

PH measures the concentration of active hydrogen ions, while titratable acidity reflects the total buffer capacity of the yogurt, including free and neutralized acids. PH and titratable acidity were measured according to the AOAC official method 942.15 (AOAC, 2000). After combining 10 g of yogurt sample with 10 mL of hot distilled water and titrating with 0.1 N NaOH while using a 0.1% phenolphthalein indicator, the titratable acidity was expressed as g/100 g of lactic acid in yogurt.

### 2.7. Determination of Water-Holding Capacity (WHC) of Yogurt Samples

A total of 20g of yogurt was centrifuged at 3000 rpm for 10 min (4 °C) (Thermo Scientific, Waltham, MA, USA). The supernatant was poured off, and the precipitation (W) was weighed.

The water-holding capacity (WHC) was calculated according to Equation (1):

(1) $$WHC=\frac{W}{Y}\times 100\%$$

where *W* presents the mass of the precipitation and *Y* presents the mass of the analyzed yogurt.

### 2.8. Texture Analysis of Yogurt Samples

TPA analysis of yogurt for hardness and adhesion was carried out using a texture analyzer (FTC, Beijing Ying Sheng Hengtai Technology Co., Ltd. Beijing, China) with an A/BE-35 probe under the following conditions: measuring speed of 2 mm/s, premeasurement speed of 1 mm/s, post measurement speed of 3 mm/s, and an inductive force of 2 g. The measurements were repeated three times for each sample.

### 2.9. Determination of Resveratrol Retention Rate

The detection of the resveratrol retention rate was carried out using a high-performance liquid chromatograph (Agilent 1260 HPLC, Santa Clara, CA, USA) and a Venusil XBP (5 μm, 250 mm × 4.5 mm) column. The UV detection wavelength was set to 306 nm; the mobile phase included acetonitrile and water (40:60, *v*/*v*); the column temperature was 25 °C; and the flow rate was 1 mL/min. The yogurt samples were diluted with anhydrous ethanol and subjected to 30 min of sonication at 25 °C to extract the resveratrol. The resveratrol retention rate during storage at 4 °C was determined as follows:

$$Retention\;rate\;(\%)=\frac{C_{n}}{C_{0}}\times 100\;$$

where *C*_0_ is the content of resveratrol in yogurt on day 0, *C_n_* represents the content of resveratrol in yogurt on day n.

### 2.10. The Bioaccessibility of Resveratrol

The digest was centrifuged at 12,000 rpm for 30 min. All the micelles were taken, and the volume was measured. Then, the micelles were diluted by adding ethanol (extracted with trichloroacetic acid (TCA) solution (7.5%, *v*/*v*)). The mixture was centrifuged at 8000 rpm for 3 min at 4 °C, and the supernatant was filtered through a 0.22 organic filter membrane and quantified by HPLC.

Bioaccessibility (%)=T/I×100

where *T* is the total resveratrol in micelles and *I* is the initial amount of resveratrol in the digestion system.

### 2.11. ^1^H NMR Spectra Acquisition of Yogurt

The NMR experiments were conducted on a Bruker AVANCE III 600 MHz NMR spectrometer equipped with a CryoProbe (Bruker Biospin, Ettlingen, Germany) with ^1^H and ^13^C frequencies of 600.13 and 150.90 MHz, respectively. ^1^H NMR spectra were acquired using a standard noesygppr1d pulse sequence with a spectral width of 20 ppm, an acquisition time of 1.36 s, and a mixing time of 100 ms. A total of 128 transients were collected into 32,000 data points. For metabolite assignments, the Human Metabolome Database (https://hmdb.ca/ (accessed on 18 February 2023)) and previous studies were referenced.

### 2.12. Data Analysis

A statistical analysis of the data was conducted using the SPSS software with a confidence level of 95%. Origin 2021 was used for data plotting. All spectra were corrected for phase and baseline using the Mestre Nova 14.2.0 software, with chemical shifts referenced to the methyl signal of TSP (δ_H_ 0.00). The spectral region between δ_H_ 1.5 and δ_H_ 5.5 was divided into bins with a width of 0.002 ppm. The integrated areas of all bins were then normalized to the dry sample weights. Multivariate data analysis was conducted on the normalized data using the software SIMCA-P+ (V13.0, Umetrics, Sweden).

## 3. Results

### 3.1. The Appearance and Particle Size of Emulsion Stablized by NaCas(NN), DGMO (DN), and NaCas/DGMO Particles (NDN)

**Figure 1:**
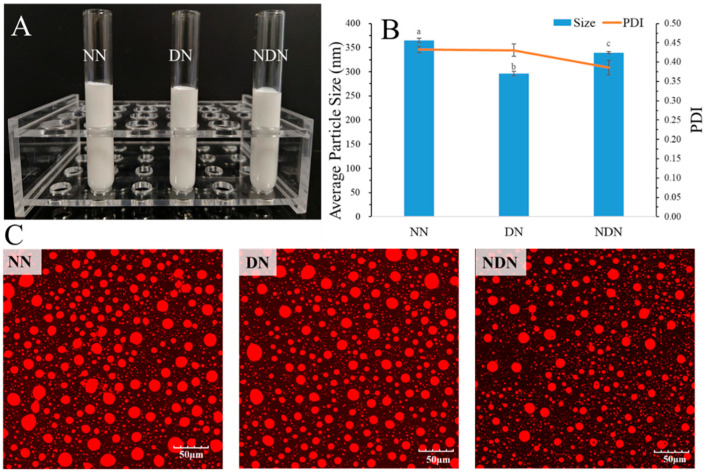
(**A**) The appearance, (**B**) average particle size, PDI, and (**C**) confocal laser scanning microscopy (LCSM) images of resveratrol-loaded emulsion stabilized by NaCas (NN), DGMO (DN), and NaCas/DGMO particles (NDN). The oil droplets stained red by Nile. Means with the different letter are significantly different. The measurements were repeated three times.

The resveratrol-loaded emulsions stabilized by NN, DN, and NDN appeared similar and were not stratified, with a milky white color and uniform dispersion (Figure 1A). The average particle size distribution of emulsion droplets showed that the three emulsions were submicron-sized, with a PDI distribution ranging from 0.38 to 0.42 (Figure 1B), which indicated the stability of the emulsions. Nile-stained CLSM images described that the droplets of the emulsion were uniformly distributed (Figure 1C). The results are consistent with our previously reported results [24].

### 3.2. The Physicochemical Properties of Yogurts

**Figure 2:**
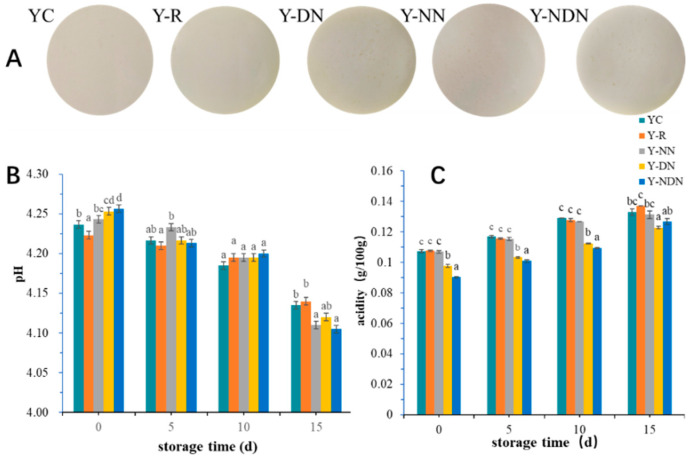
(**A**) Appearance, (**B**) the pH, and (**C**) the titratable acidity of five formulated yogurts, including YC, Y-R, Y-NN, Y-DN, and Y-NDN, at 0, 5, 10, and 15 days, respectively (The data are represented as mean ± SD, *n* = 3. Means with the different letter are significantly different, and means with at least one common letter are not significantly different.).

The emulsions containing resveratrol, stabilized by NN, DN, and NDN, were added to yogurts. The control groups were normal yogurt and yogurt added with free resveratrol. The addition of resveratrol and resveratrol-loaded emulsions barely impacted the appearance of the yogurts, as shown in Figure 2A. As depicted in Figure 2B, the pH values of Y-NN, Y-DN, and Y-NDN slightly increased in comparison to YC’s initial pH value of 4.24 at day 0. The pH values of the five groups of yogurts decreased over time, though the decrease was not significant. So, compared to YC, the treated groups showed a decrease in yogurt acidity with storage time, especially for the Y-NN and Y-NDN groups. All the titratable acidities of five groups of yogurts showed an upward trend (Figure 2C). Table 2 displays changes in the physicochemical properties of yogurts during storage. The quality of yogurt was evaluated through typical indicators such as hardness, chewiness, gumminess, cohesiveness, and resilience.

**Figure 3:**
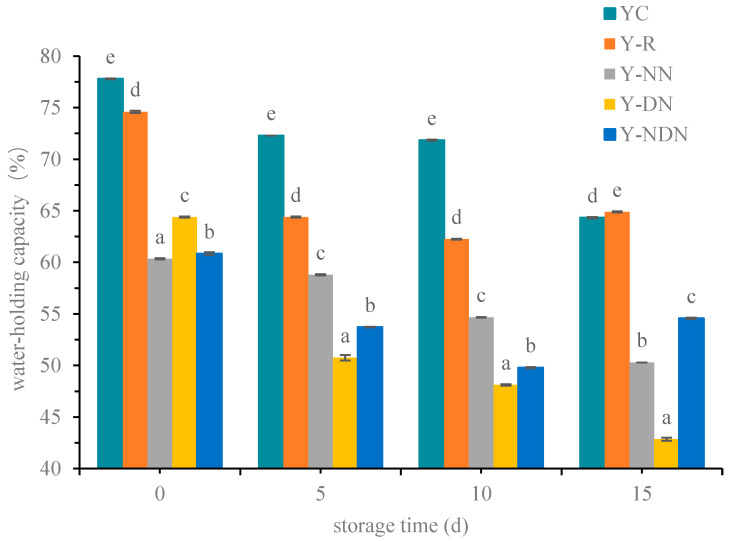
The water-holding capacity (WHC) of five groups of yogurts (YC, Y-R, Y-NN, Y-DN, and Y-NDN) at day 0, day 5, day 10, and day 15, respectively (The data are represented as mean ± SD, *n* = 3. Means with the different letter are significantly different.).

The WHC values of Y-R, Y-NN, Y-DN, and Y-NDN were 74.6 ± 0.1%, 60.35 ± 0.05%, 64 ± 0.05%, and 60.85 ± 0.15%, respectively, which were smaller than those of YC at the initial storage, as shown in Figure 3. Among the composite emulsion yogurt samples, Y-DN exhibited the strongest water-holding capacity. The WHC of all groups decreased over the 15-day storage period. The WHC of complex emulsion yogurt was consistently and significantly lower than that of YC and Y-R.

### 3.3. Retention of Resveratrol in Yogurt Samples

**Figure 4:**
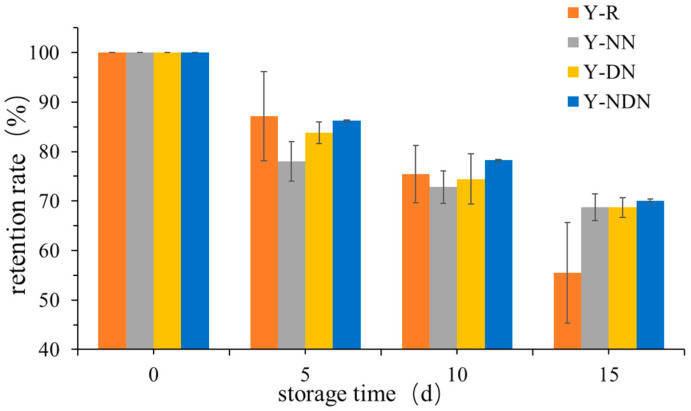
The retention rate of four groups of yogurts (Y-R, Y-NN, Y-DN, and Y-NDN at day 0, day 5, day 10, and day 15, respectively) (mean ± SD, *n* = 3).

The retention rate of resveratrol on day 0 was considered 100%. As illustrated in Figure 4, the retention of resveratrol in the form of composite emulsion was significantly better compared to its free form after the 15-day storage period. The retention rate of resveratrol differed across the four samples, with Y-NDN retention being the highest (70.05 ± 0.40%) and Y-NN, Y-DN, and Y-R retention being lower (68.81 ± 1.10%, 68.82 ± 2.01%, and 55.80 ± 6.67%, respectively). The retention rate of resveratrol declined over time during storage.

### 3.4. Bioaccessibility of Resveratrol in Yogurt Samples

**Figure 5:**
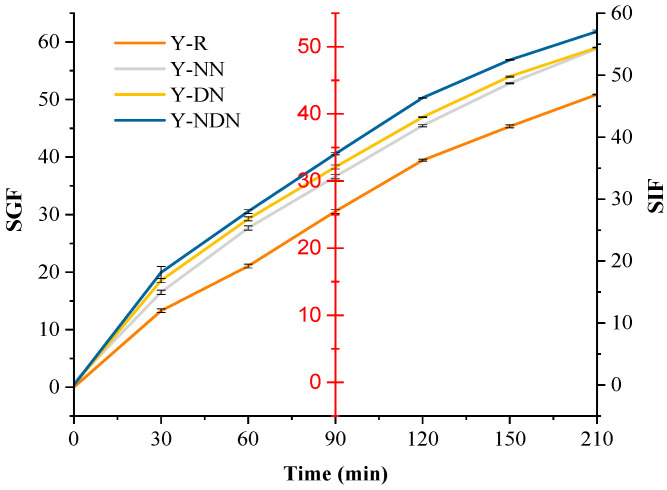
The dynamic bioaccessibility of resveratrol in four formulated yogurts (Y-R, Y-NN, Y-DN, and Y-NDN) during the digestion process of the gastrointestinal simulated fluid; among them, 0–90 min were simulated in the gastric fluid digestion period, and 90–210 min were simulated in the intestinal fluid digestion period. (mean ± SD, *n* = 3).

Figure 5 displays the resveratrol bioaccessibility results of Y-R, Y-NN, Y-DN, and Y-NDN, being 50.86%, 49.7%, 54.45%, and 57.05%, respectively, after in vitro gastrointestinal digestion. There was a significant alteration in the resveratrol bioaccessibility between each group (*p* < 0.05), and the Y-NDN group showed the highest resveratrol bioaccessibility.

### 3.5. Change in Yogurt Nutritional Profile before and after In Vitro Digestion

#### 600 MHz ^1^H NMR Spectra of Yogurts

**Figure 6:**
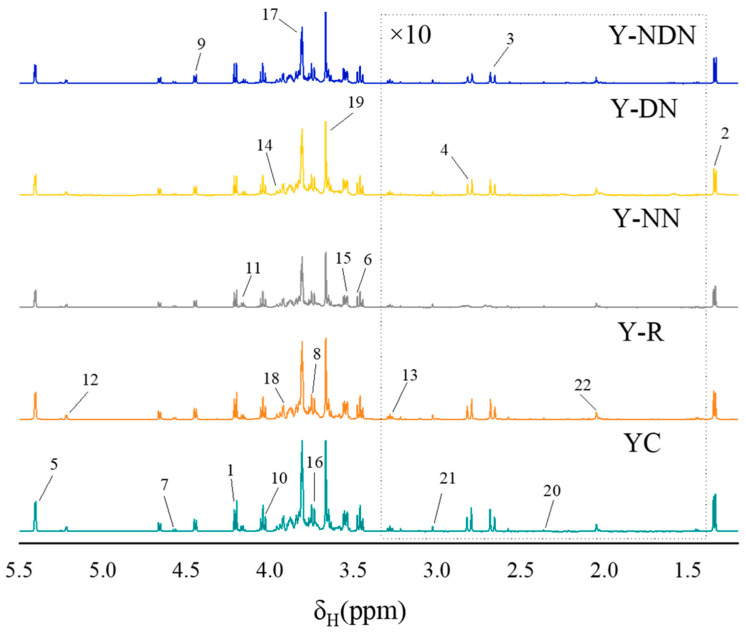
Typical 600 MHz ^1^H NMR spectra of undigested yogurt from YC, Y-R, Y-NN, Y-DN, and Y-NDN on day 0. The region of δ_H_ 1.4–δ_H_ 3.3 ppm was vertically magnified by 10 times, and the signals are assigned in Table 3.

The changes in nutritional metabolites were detected through ^1^H-NMR. As shown in Figure 6, the peaks at δ1.23 and δ2.2 ppm were assigned to acyl chains of milk fat, while those at 1.33 ppm were assigned to lactic acid. The weak signals at δ2.2 to δ3.1 ppm were considered the methyl or methylene groups of citrate, creatine, and lecithin, which were minor constituents of yogurt. The signals detected from δ3.1 to δ5.1 ppm were from D-lactose and D-galactose. However, these signals overlapped strongly. The region between the chemical shifts of δ5.1 to δ5.4 ppm exhibited a few feeble signals that were assigned to the amide protons of the protein. The primary proteins present in cow’s milk were casein and lactoglobulin [28,29]. Table 3 summarizes the ^1^H-NMR signal assignments, the corresponding functional groups, and the chemical shifts of metabolites. The stacking spectra of five groups of freshly prepared yogurts before digestion indicated that the metabolites of yogurt are basically similar.

**Figure 7:**
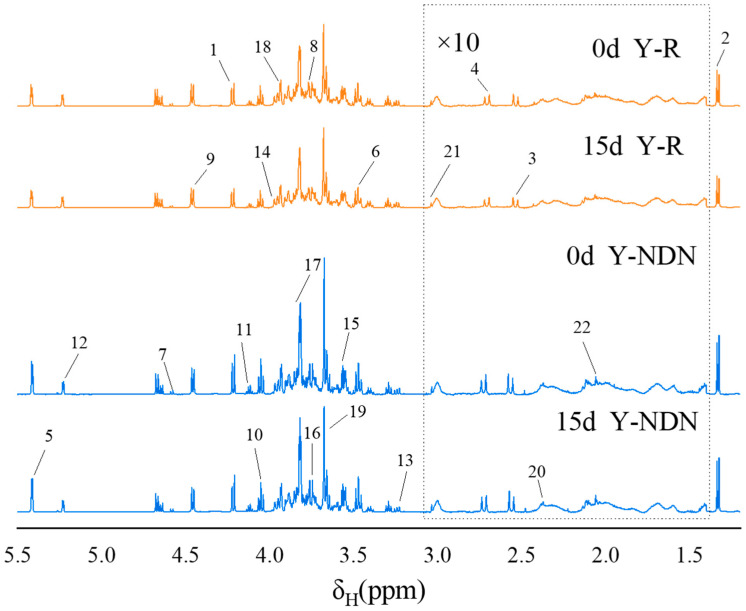
Typical 600 MHz ^1^H NMR spectra of Y-R and Y-NDN yogurts after digestion on day 0 and day 15. Simultaneously expand the peak area of δ_H_ 1.4–δ_H_ 3.3 ppm by 10 times; the numbered signals are depicted in Table 3.

The stacking spectra of Y-R and Y-NDN at 0 and 15 days after digestion are displayed in Figure 7. When compared to Figure 6, it can be observed that different yogurt groups exhibited different post-digestion modifications in addition to changes to specific metabolites. Following digestion, the Y-NDN group had a more notable shift in the composition of several metabolites.

**Figure 8:**
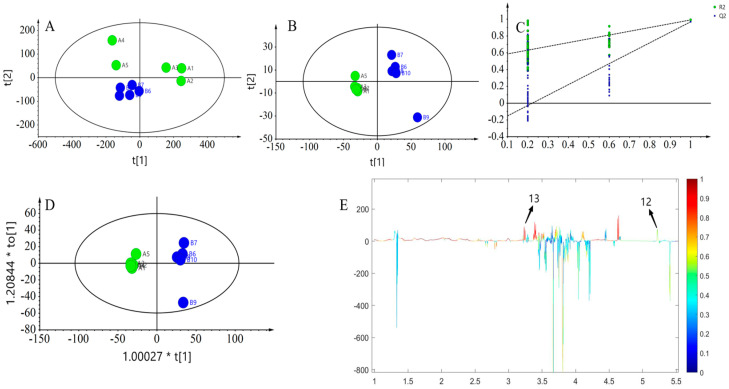
Multivariate analysis of the five yogurts before and after digestion at day 0: (**A**) cross-validated PCA score plot (R^2^X = 0.898, Q^2^ = 0.792); (**B**) cross-validated PLS-DA score plot (R^2^X = 0.72, Q^2^ = 0.97); (**C**) permutation experiment of the PLS-DA model (number of permutations *n* = 200); (**D**) OPLS-DA score plot for yogurt (R^2^X = 0.819, Q^2^ = 0.971, *p* = 0.003); and (**E**) corresponding correlation coefficient loading plot (R^2^X = 0.72, Q^2^ = 0.933). The green circle represents the five groups of formulated yogurts on day 0, while the blue circle represented the digesta of five groups of yogurts after in vitro digestion on day 0.

**Figure 9:**
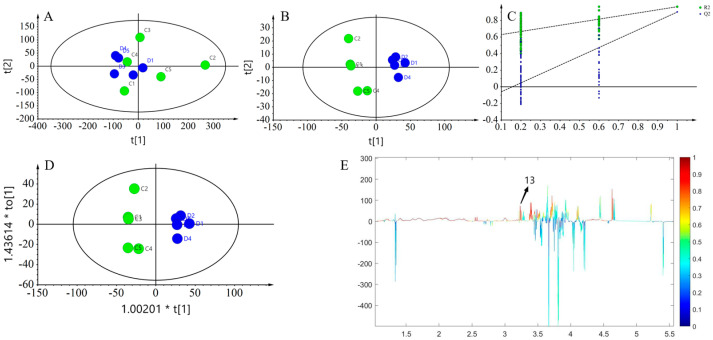
Multivariate analysis of the five yogurts before and after digestion at day 15: (**A**) cross-validated PCA score plot (R^2^X = 0.918, Q^2^ = 0.737); (**B**) cross-validated PLS-DA score plot (R^2^X = 0.7, Q^2^ = 0.901); (**C**) permutation experiment of PLS-DA model (number of permutations *n* = 200); (**D**) OPLS-DA score plot for yogurt (R^2^X = 0.7, Q^2^ = 0.902, *p* = 0.009); and (**E**) corresponding correlation coefficient loading plots (R^2^X = 0.72, Q^2^ = 0.933). The green circle represents the five groups of formulated yogurts on the 15th day, while the blue circle represents the digesta of five groups of yogurts after in vitro digestion on the 15th day.

The ^1^H NMR profiles of five groups of yogurts before and after 0 and 15 days of digestion, respectively, were subjected to multivariate statistical analyses to explore the changes in the nutrient compositions of the yogurts before and after digestion. It could be seen from the PCA score plots (Figure 8A) that the yogurts showed an aggregated state within the groups before and after digestion, and obvious distinctions were found between the groups. This indicated that the addition of a resveratrol complex emulsion did not affect the quality of the yogurt, but compared to pre-digestion, the nutritional composition of the yogurt in the post-digestion groups appeared significantly different. The PLS-DA model score plots (Figure 8B,C) showed that the model was established successfully, which indicates that the changes in the nutritional composition before and after digestion of yogurt are significant. In addition, the trend of differentiation between yogurt groups was more evident from the OPLS-DA score plot (Figure 8D), with a *p* value of CV-ANOVA less than 0.05. The results of the analyses before and after 15 days of digestion (Figure 9) were similar to those after 0 days of digestion, with clustering within yogurt groups and differentiation between groups. Compared with before and after 0-day digestion, the intergroup clustering of yogurt groups was slightly inconspicuous, though the multivariate statistical analysis showed that the model was valid. In addition, the multivariate statistical analysis of the yogurt groups before digestion at 0 and 15 days showed that the PLS-DA permutation test model was invalid, indicating that the nutrient composition of yogurt stored for 15 days was almost unchanged compared with that stored for 0 days and that the composite emulsion yogurt was still very stable and its nutrient composition was better preserved during the shelf life. The results of intergroup difference metabolites in the correlation coefficient loading plots of the models before and after yogurt digestion (Figure 8E and Figure 9E) are shown in Table 4 and Table 5. This was consistent with a previous report that demonstrated that the addition of composite emulsion can significantly change some metabolites in yogurt and improve its nutritional value.

As shown in Table 4 and Table 5, a significant increase in choline and N-acetyl-D-glucosamine in yogurt occurred after digestion on day 0 and day 15. 

## 4. Discussion

In order to comprehensively evaluate resveratrol-fortified yogurt, we measured the basic physicochemical properties of five groups of yogurts. During storage, the main sugar in yogurt, lactose, is converted into lactic acid by *Lactobacillus*, resulting in changes to the pH and acidity that reflect the basic physical and chemical properties of yogurts. Hence, it was crucial to examine the alterations in the pH and acidity that occurred during yogurt storage [30]. The commercial yogurt had an optimal pH range of 4.00–4.40, with a suggested pH range of 4.00–4.25 for improved taste. During storage for 15 days, the pH of all the yogurt samples remained between 4.00 and 4.26, preserving the taste of the yogurt samples (Figure 2A).

On the 15th day, a marked reduction in pH was observed in Y-NDN compared to YC. This indicated that post-storage acidification occurred obviously in the Y-NDN yogurt, resulting from the production of organic acids from lactose fermentation [11,14]. The result was consistent with the change in titratable acidity, which was related to the production of D-lactic acid by *Lactobacillus bulgaricus* [31,32]. The acidity was generally increased in five groups of yogurts during the 15-day storage period, and the value in the Y-NDN group was lower than the control on day 15 (Figure 2B). This suggested that the addition of resveratrol emulsion had an effect on the growth and metabolism of *Lactobacillus*, resulting in the titratable acidity of yogurt with resveratrol emulsion being lower than the control yogurt during later storage [33]. This was consistent with the findings of Settachaimongkon [34].

Since yogurt is a commodity on the market, consumer preferences impact its popularity and sales volume, making the sensory assessment of the product important. Y-R and Y-NN were harder than YC, whereas Y-DN and Y-NDN were softer. Notably, as the storage time increased, the hardness of each yogurt group initially increased and then declined. Conversely, chewiness decreased first and then rose over time. Chewiness was influenced not only by hardness but also by cohesiveness and resilience. Throughout storage, both cohesion and elasticity decreased initially and then increased, further impacting chewiness. Gumminess appeared to have minimal variations in each group (Table 2). There were slight disparities in the transformations of the physicochemical properties [35]. This implied that the added emulsion had no negative effect on the taste of the yogurt, which supported the feasibility of the yogurt formulation.

Yogurt’s water-holding capacity (WHC) could serve as an indirect indicator of its texture. WHC refers to the amount of water retained after centrifugation. A larger WHC indicates a higher water retention capacity, which enables yogurt to bind with more water molecules, resulting in less whey precipitation in the final product [36]. The results indicated that the addition of emulsion affected the WHC of yogurt throughout the storage period. The decreased stability and WHC of yogurt might be attributed to the weakening of the affinity linkage between protein molecules in the gel system as the acidity increased during storage (Figure 3). This in turn led to the loosening of the protein network structure formed by gel agglomeration, resulting in a decrease in the stability and water-holding capacity of yogurt [37]. Furthermore, the process of diluting the emulsion complex of resveratrol with water during preparation led to a reduction in the protein concentration. It resulted in a decrease in the water-holding ability of yogurt. Among these, Y-NDN minimally affected YC’s water-holding capacity, and the water retention rate continued to rise over the 15 days. This could be attributed to the hydration of triglycerides in rice bran oil within NaCas-DGMO nano-emulsions, interacting with lipid head groups to create a more stable liquid crystal structure. Consequently, yogurt’s water-holding ability improved by enhancing its capacity to retain and bind water [38].

The retention rate of resveratrol in yogurt was associated with its nutritional value, which was also closely related to the shelf life of yogurt. Since resveratrol was susceptible to photooxidative degradation over time, it was necessary to measure the retention rate of resveratrol. The retention rate of resveratrol in four groups of yogurts showed a decreasing trend over time (Figure 4). Approximately 70% of the resveratrol was still retained in the Y-NDN yogurt at the end of storage, which might be due to the lowest pH levels in Y-NDN after 15 days of storage, and the rate of resveratrol degradation was affected by pH [39]. Therefore, the NaCas-DGMO emulsion effectively improved the retention rate of resveratrol in yogurt during storage to preserve the nutritional value of yogurts.

Additionally, we also explored the changes in the nutritional profile of resveratrol-fortified yogurt after in vitro digestion. Bioaccessibility refers to the proportion of nutrients absorbed that, after digestion in the gastrointestinal tract, enter the body’s systemic circulation and are available for normal physiological functions [40]. The yogurt samples with the NaCas-DGMO emulsion exhibited a greater enhancement in resveratrol bioaccessibility when compared to other composite emulsions (Figure 5). It was attributed to the elevated resistance of the stable emulsion of oil to lipid digestion. The stable emulsion resisted lipid digestion due to its elevated resistance. The tightly packed molecular structure at the oil–water interface blocked spatial sites, hindering bile salts and pancreatic enzymes’ displacement. This reduced lipase’s ability to displace lipid droplets, thereby decreasing the release of resveratrol for digestion and degradation in the aqueous phase [41,42]. NaCas/DGMO emulsions enhanced the thickness and density of the interfacial layer by modifying a single interfacial layer. They also prevented the diffusion of bile acid salts, Ca^2+^, and pancreatic lipase at the interface and regulated the release properties of active ingredients from lipid carriers [43]. Meanwhile, the hydrolysis of rice bran oil produced free fatty acids during small intestine digestion, which contributed to the formation of mixed micelles. It enhanced the dissolution of hydrophobic molecules in the small intestine fluid and increased their bioaccessibility.

In terms of the nutritional components in the five groups of yogurts, the amount of D-maltose, lactose, lactic acid, and other substances in YC was slightly higher than in other groups before digestion on day 0 (Figure 8). This indicates that the addition of resveratrol emulsion did not negatively affect the fundamental properties of the yogurt and preserved the original nutritional components of the yogurt. Compared to their undigested forms, both Y-NDN and Y-R exhibited variations in many nutritional compounds after digestion. The increased levels of essential amino acids and the breakdown of lactose may suggest potential improvements in digestion and absorption [44], which could translate to physiological benefits in an in vivo setting, meriting further research. The increase in glucose levels after digestion to some extent indicates a decrease in the tissue utilization of glucose, which may be due to effective improvements in glucose tolerance by resveratrol [45]. Lactate was transformed from pyruvate through the metabolism of the gut microbiota. Resveratrol inhibited the absorption of glucose by lactate dehydrogenase (LDH), which lowered both the consumption of glucose and the absorption of lactate [46].

Based on the properties of resveratrol, we specifically compared the differences in nutritional components between Y-R and Y-NDN after digestion (Figure 9). After digestion, many nutritional components of the two groups increased, although compared to Y-R, the peak areas of lactic acid, choline, glucose, N-acetyl-D-glucosamine, and other substances in Y-NDN were significantly higher. This indicated a pronounced increasing trend, which may have been caused by the higher stability of the resveratrol composite emulsion compared to free resveratrol. It can be seen that choline and N-acetyl-D-glucosamine showed significant changes before and after digestion from the correlation coefficient loading plots (Table 4).

N-acetyl-D-glucosamine was derived by the substitution of acetylamino groups on the C2 hydroxyl group in glucose molecules and participated in the synthesis of glycoproteins. It also promoted the growth of *Bifidobacteria* and *Lactobacillus* and regulated the distribution of the gut microbiota. The effects of resveratrol on acetyl glucosidase might cause an increase in N-acetyl-D-glucosamine following digestion. Since the stability of a resveratrol composite emulsion was superior to that of free resveratrol, the N-glycoprotein concentration in the Y-NDN group was higher than that of the Y-R group. Chitosan was composed of the monomer N-acetyl-D-glucosamine. Some studies have indicated that incorporating chitosan into yogurt may influence nutrient absorption and provide dietary fiber [47]. An increase in N-acetyl-D-glucosamine content could promote the synthesis of chitosan to achieve high nutrient availability.

Choline, a crucial water-soluble micronutrient, was essential for maintaining cell structure and function and for the synthesis of the neurotransmitter acetylcholine. It was a key precursor in the synthesis of phosphatidylcholine, nerve sphingomyelin, and carboxylated aldehyde phospholipids. Furthermore, it could be helpful for improving fatty liver disease and cirrhosis of the liver [48]. Our findings revealed that the addition of resveratrol to yogurt had an effect on the choline levels. This was probably induced by resveratrol, which had an inhibitory effect on acetyl esterase, one of the precursors of acetylcholine synthesis, thus slowing the tendency to reduce choline levels [49]. The choline levels in yogurt were notably elevated post-digestion, which promoted nutrient absorption, growth, and development and improved immunity. The study on fermented resveratrol-enriched yogurts confirmed that resveratrol-enriched yogurt’s choline content increased significantly after digestion.

## 5. Conclusions

In the present study, resveratrol-loaded emulsions were effectively applied to yogurts. The impact of resveratrol with different formulations on the physicochemical properties, including pH, titratable acidity, water-holding capacity, and retention rate of resveratrol, was characterized for 15 days of storage at 5-day intervals. It was found that the addition of resveratrol emulsion reduced the hardness of yogurt, while titratable acidity and water-holding capacity remained the same. The stability of resveratrol added as an emulsion was significantly higher than that of the free form. Furthermore, in vitro digestion showed that encapsulation effectively improved the dynamic bioaccessibility of resveratrol in a sustained manner. The NMR-based analysis of the nutritional profile before and after in vitro digestion showed that the nutritional fortification of resveratrol emulsion promoted the release of nutrients, thereby improving the nutritional value of yogurt.

## Figures and Tables

**Table 1:** The composition of five types of yogurts.

Sample	NaCas (g/100 g)	DGMO (g/100 g)	Rice Bran Oil (g/100 g)	Resveratrol (g/100 g)	Water (g/100 g)
Y-C	-	-	-	0.0075	15
Y-R	-	-	-		
Y-NN	0.135	-	1.364		
Y-DN	-	0.135	1.364		
Y-NDN	0.0675	0.0675	1.364		

**Table 2:** Textural parameters of five formulated yogurts, YC, Y-R, Y-NN, Y-DN, and Y-NDN, at day 0, day 5, day 10, and day 15.

Sensory	Days	YC	Y-R	Y-NN	Y-DN	Y-NDN
Hardness/gf	0	1.669 ± 0.001 ^a^	1.597 ± 0.074 ^a^	2.124 ± 0.059 ^a^	1.645 ± 0.177 ^a^	1.580 ± 0.289 ^a^
	5	2.723 ± 0.048 ^b^	3.475 ± 0.039 ^c^	2.917 ± 0.128 ^bc^	1.871 ± 0.005 ^a^	1.845 ± 0.404 ^a^
	10	3.160 ± 0.074 ^b^	4.008 ± 0.039 ^c^	3.404 ± 0.297 ^b^	1.812 ± 0.159 ^a^	1.734 ± 0.023 ^a^
	15	1.673 ± 0.17 ^abc^	1.973 ± 0.007 ^bc^	2.269 ± 0.353 ^c^	1.043 ± 0.455 ^ab^	0.875 ± 0.142 ^a^
Chewiness/gf	0	1.016 ± 0.207 ^a^	1.414 ± 0.069 ^a^	2.870 ± 0.045 ^a^	2.412 ± 1.184 ^a^	1.959 ± 1.110 ^a^
	5	0.497 ± 0.067 ^a^	0.709 ± 0.709 ^a^	0.576 ± 0.174 ^a^	1.700 ± 0.322 ^a^	0.591 ± 0.306 ^a^
	10	0.989 ± 0.022 ^a^	2.045 ± 0.093 ^b^	1.194 ± 0.254 ^a^	2.307 ± 0.351 ^b^	1.172 ± 0.006 ^a^
	15	2.324 ± 1.306 ^a^	2.871 ± 0.972 ^a^	1.222 ± 0.257 ^a^	2.383 ± 1.864 ^a^	0.319 ± 0.056 ^a^
Gumminess/gf	0	1.388 ± 0.091 ^a^	1.424 ± 0.060 ^a^	2.870 ± 0.045 ^a^	2.412 ± 1.184 ^a^	2.154 ± 0.914 ^a^
	5	2.081 ± 0.015 ^a^	1.565 ± 1.565 ^a^	2.248 ± 0.200 ^a^	1.856 ± 0.166 ^a^	2.392 ± 1.196 ^a^
	10	2.797 ± 0.106 ^b^	3.695 ± 0.195 ^c^	2.899 ± 0.079 ^b^	2.307 ± 0.351 ^b^	1.323 ± 0.016 ^a^
	15	2.403 ± 1.227 ^a^	2.871 ± 0.972 ^a^	2.811 ± 1.133 ^a^	2.383 ± 1.864 ^a^	0.442 ± 0.068 ^a^
Cohesiveness/gf	0	0.832 ± 0.055 ^a^	0.892 ± 0.004 ^a^	1.353 ± 0.058 ^a^	1.405 ± 0.569 ^a^	1.301 ± 0.341 ^a^
	5	0.765 ± 0.019 ^a^	0.456 ± 0.456 ^a^	0.775 ± 0.103 ^a^	0.992 ± 0.086 ^a^	1.213 ± 0.383 ^a^
	10	0.885 ± 0.013 ^a^	0.921 ± 0.040 ^a^	0.860 ± 0.098 ^a^	1.300 ± 0.308 ^a^	0.763 ± 0.001 ^a^
	15	1.377 ± 0.594 ^a^	1.454 ± 0.488 ^a^	1.190 ± 0.314 ^a^	1.860 ± 0.977 ^a^	0.505 ± 0.005 ^a^
Resilience/gf	0	0.603 ± 0.156 ^a^	0.588 ± 0.072 ^a^	0.812 ± 0.067 ^a^	0.447 ± 0.236 ^a^	0.685 ± 0.014 ^a^
	5	0.387 ± 0.133 ^a^	0.376 ± 0.035 ^a^	0.269 ± 0.070 ^a^	0.360 ± 0.123 ^a^	0.345 ± 0.166 ^a^
	10	0.308 ± 0.004 ^ab^	0.294 ± 0.001 ^a^	0.282 ± 0.023 ^a^	0.720 ± 0.061 ^c^	0.427 ± 0.040 ^b^
	15	0.252 ± 0.132 ^a^	0.509 ± 0.087 ^a^	0.202 ± 0.072 ^a^	0.589 ± 0.140 ^a^	1.202 ± 0.678 ^a^

Data are represented as mean ± SD, *n* = 3. Means with the different letter are significantly different, and means with at least one common letter are not significantly different.

**Table 3:** Metabolite NMR signal assignments of aqueous phase extracts from differently formulated yogurts.

Number	Compound Name	Functional Group	Chemical Shift (ppm)
1	L-Threonine	αCH	3.52 (d) ^a^
		βCH	4.17 (m)
		γCH_3_	1.30 (d)
		CH_2_	4.07 (m)
2	Lactic acid	α-CH	4.08 (q)
		β-CH_3_	1.33 (d)
3	Citric acid	1CH	2.66 (d)
		3CH	2.58 (d)
4	Lecithin	1CH_2_	2.81 (d)
		2CH	3.89 (t)
5	D-Maltose	1CH_2_	3.85 (m)
		2CH	3.70 (m)
		4CH	3.68 (m)
		6CH	5.37 (d)
		8CH	3.94 (m)
		9CH_2_	3.46 (dd)
		10CH	5.21 (d)
		12CH_2_	3.79 (m)
		12CH_2_	3.92 (dd)
6	α-D-Glucose	2CH	3.58 (dd)
		3CH	3.80 (dd)
		4CH	3.49 (dd)
		5CH	3.91 (dt)
		6CH	3.85 (m)
7	β-D-Glucose	1CH_2_	3.76 (d)
		2CH	3.49 (dt)
		3CH	3.40 (dd)
		4CH	3.50 (dd)
		5CH	3.28 (dd)
		6CH	4.61 (d)
8	α-D-galactose	1CH_2_	3.77 (quint)
		2CH	3.78 (q)
9	β-D-galactose	1CH	4.57 (d)
		2CH	3.49 (m)
10	Lactose	2CH	5.18 (td)
		3CH	5.37 (d)
		5CH	4.12 (dd)
		6CH	3.87 (dd)
		8CH	3.61 (dd)
		9CH	3.90 (dd)
		10CH	4.02 (dd)
		11CH	3.86 (td)
		12CH_2_	3.75 (d)
11	α-D-Ribose	1CH	5.40 (d)
		2CH	4.12 (q)
12	N-Acetyl-D-Glucosamine	3CH	4.44 (dd)
		5CH	3.44 (dd)
			5.22 (dd)
13	Choline	1CH_3_	3.22 (s)
14	Histidine	2CH	3.98 (dd)
15	Glycine	1CH_2_	3.55 (s)
16	Lysine	5CH_2_	3.02 (t)
		1CH	3.75 (t)
17	Glycerol	1CH_2_	3.60 (dd)
		2CH	3.84 (m)
18	Sorbitol		3.82 (dd)
		3CH	3.84 (dd)
			3.94 (ddd)
19	Sucrose		3.69 (s)
			4.22 (d)
			5.42 (d)
20	Malic acid		2.36 (dd)
		1CH	2.68 (dd)
		2CH	4.31 (dd)
21	Creatine	2CH_3_	3.02 (s)
22	N-acety-glycoproteins	CH_3_	2.05 (s)

^a^ represents the multiplicity; s, singlet; d, doublet; t, triplet; q, quartet; m, multiplet; dd, double doublet.

**Table 4:** Correlation coefficients for metabolites that significantly changed in the OPLS-DA model for yogurt before and after digestion on day 0.

Number	Metabolite	δ^1^H (ppm)	Correlation Coefficient (r) ^a^
12	N-acetyl-D-glucosamine	5.219	0.842
13	Choline	3.225	0.883

^a^ The critical value for |r| is 0.81, *p* < 0.05.

**Table 5:** Correlation coefficients for metabolites that significantly changed in the OPLS-DA model for yogurt before and after digestion on day 15.

Number	Metabolite	δ^1^H (ppm)	Correlation Coefficient (r) ^a^
13	Choline	3.225	0.932

^a^ The critical value for |r| is 0.81, *p* < 0.05.

## Data Availability

Data is contained within the article.
